# Antioxidant and Antimicrobial Properties of Semi-Processed Frozen Prickly Pear Juice as Affected by Cultivar and Harvest Time

**DOI:** 10.3390/foods9020235

**Published:** 2020-02-22

**Authors:** Rosa Palmeri, Lucia Parafati, Elena Arena, Emanuela Grassenio, Cristina Restuccia, Biagio Fallico

**Affiliations:** Di3A, Dipartimento di Agricoltura, Alimentazione e Ambiente, University of Catania, via S. Sofia 100, 95123 Catania, Italy; rpalmeri@unict.it (R.P.); lucia.parafati@unict.it (L.P.); earena@unict.it (E.A.); emanuelagrassenio@gmail.com (E.G.); bfallico@unict.it (B.F.)

**Keywords:** betalains, antibacterial activity, antioxidant activity, differently colored cultivars, food ingredient

## Abstract

Consumer interest toward natural ingredients is creating a growing trend in the food industry and research for the development of natural products such as colorants, antimicrobials and antioxidants. Semi-processed frozen prickly pear (*Opuntia ficus-indica* (L.) Mill.) juices (PPJs), obtained from three cultivars with white, red and yellow pulp, with different harvest times (“Agostani” and “Bastardoni”), were characterized for betalains, total phenolics, flavonoids, carotenoids, antioxidant capacity (by 2,2-diphenyl-1-picryl-hydrazyl-hydrate (DPPH)) and antimicrobial potential against foodborne pathogenic bacteria. Through chemical assays, PPJs showed high contents of total polyphenols, flavonoids and betalains and marked antioxidant capacity. PPJs from the first harvest (“Agostani”) revealed the significantly highest amount of polyphenols in white cultivar and of betacyanin and betaxanthin in yellow and red cultivars; antioxidant capacity was significantly higher in “Agostani” PPJs than in “Bastardoni” ones. Regarding antimicrobial potential, all PPJs revealed good antibacterial activity, particularly against *Salmonella enterica* as evidenced by the widest inhibition haloes. These results encourage the suitability of the first flowering prickly pear fruits, with low market value as fresh fruit but with high nutritional features, to be processed as semi-finished product. In particular, its use as ingredient in foods with high risk of *Salmonella* contamination may act as a natural preservative.

## 1. Introduction

Prickly pear (*Opuntia ficus-indica* (L.) Mill.), native to Central America, is today cultivated in arid and semi-arid region of Southern Europe, the Middle East and northern Africa. In Sicily (Italy) the prickly pear fruits are mainly traded and consumed as fresh product. The fruits of the first plant flowering, known as “Agostani” for the fact that they ripen in August, have a limited commercial market value due to their small size and low juice content. For this reason, agronomic practices are increasingly directed to the production of large-sized fruits also called “Bastardoni”. These fruits derive from a practice called “scozzolatura”, which consists of cutting off in May the first plant flowering. Due to this process, the plant is forced into a second, even more abundant, flowering that takes place in full summer (July/August), effectively delaying its fruiting season during the fall season, thus producing juicy plump fruits.

The prickly pear fruit can be considered a healthy fruit, which is a property that has been attributed to its bioactive compounds, mainly represented by polyphenols, flavonoids and pigments such as betalains. Other authors reported the presence of vitamin C, carotenoids and taurine [1,2,3,4]. This fruit showed to be rich in polyphenolic compounds with a more antioxidant effect than vitamins. Several polyphenolic compounds were identified in prickly pear pulp mainly represented by ferulic acid derivative, rutin, gallic acid and catechin [5,6]. Vitamin C contributes to the antioxidant properties of the prickly pear juice, and it ranges between 20 and 38 mg/100 g juice. “Bastardoni” have the highest levels of vitamin C (31–38 mg/100 g) probably due to favorable seasonal conditions and among the cultivars the yellow one contains the largest amount of ascorbic acid [7]. The presence of taurine is disputed [3,8].

A recent study also demonstrated in vitro antioxidant activity of cladode extracts from Sicilian *O. ficus-indica* and recommends their novel applications in the field of nutraceutical products [9]. The betalains are water-soluble pigments that in in vivo and in vitro studies proved to be a promising alternative to supplement therapies in oxidative stress-, inflammation- and dyslipidemia-related diseases such as stenosis of the arteries, atherosclerosis, hypertension and cancer, among others [10]. Two betalainic derivatives are present in prickly pears: betacyanin, responsible for its purplish-red color; and betaxanthine, responsible for its yellowish-orange color. These pigments have demonstrated beneficial effects in the redox-regulated pathways implicated in cellular growth and inflammation, and no toxic effects have been observed in humans [4,11,12].

On the basis of such nutraceutical properties, prickly pear fruits, and in particular “Agostani” that have limited commercial value, could be processed to value-added products, such as juice, puree and concentrate, to be widely used in preparation of beverages, ice cream, yogurt, milkshakes, jams, jellies, smoothies and many other food products.

The aim of the present study was the assessment of the bioactive compounds and antioxidant capacity of semi-processed prickly pear juices (PPJs) obtained from fruit belonging to cultivars Muscaredda (white), Sanguigna (red) and Sulfarina (yellow) harvested in different period (“Agostani” and “Bastardoni”). Moreover, the potential of PPJs to act as a natural antimicrobial ingredient was assessed against Gram positive and negative foodborne pathogenic bacteria.

## 2. Materials and Methods

### 2.1. Plant Material

Frozen (−20 °C) prickly pear fruit (*Opuntia ficus-indica* (L.) Mill.) juices (PPJs) have been provided by the farm “Azienda Agricola Grassenio Gaetano”, located in San Cono (Catania, Italy). Within 24 h of the harvesting, 100 kg of fresh fruit for each cultivar have been washed and manually peeled; peeled fruits were cold-pressed and the resulting juice was packed in 1 kg polypropylene bins and immediately frozen at −20 °C.

The samples counted of three different cultivars, *Bianca* or *Muscaredda*, *Sanguigna* and *Sulfarina* characterized by white, red and yellow pulp, respectively. Prickly pear fruits, cultivated in C.da Cimia, Mazzarino (CL, Italy; geographic coordinates: 37°16′35.7′′ N 14°21′49.2′′ E and 37°16′27.2′′ N 14°21′53.7′′ E), were harvested at the end of August (“Agostani”) and in late-October (“Bastardoni”; throughout the manuscript referred as first and second harvest, respectively).

### 2.2. Physical and Chemical Analyses

All the above mentioned frozen PPJ samples were analyzed for pH, free acidity, soluble solids, dry matter and protein content.

The pH was measured using a Eutech pH 700 Meter (Thermo Fisher Scientific Inc., Waltham, MA, USA) and by placing a pH probe (Eutech pH 700 Meter) directly in the sample to be analyzed.

The free acidity, expressed as citric acid (%) was determined on 10 mL of the sample homogenized with 20 mL of distilled water by titrating with NaOH (0.1 N) up to pH 8.1.

The soluble solid content of each sample was determined using a refractometer and expressed as °Brix; moreover, the content of reducing sugar was analyzed by using the Fehling’s solution method [13].

The dry matter percentage (DM%) was determined by drying the sample at 105 °C until constant weight with an electronic moisture balance (Eurotherm, Gibertini^®^, Novate Milanese, Italy).

Protein and fat were determined using standard AOAC methods [14].

All the analyses were carried out in triplicate and results were expressed as mean ± standard deviation.

### 2.3. Bioactive Compounds and Antioxidant Activity

The PPJ samples were centrifuged at 10,000× *g* at 4 °C for 10 min in a centrifuge ALC 4239R (ALC, Winchester, VA, USA), the supernatant was filtered by a 0.45 μm pore size membrane filter (Millipore^®^, Burlington, MA, USA) and used for total polyphenols, flavonoids and DPPH determination. To evaluate betacyanin and betaxanthin content, 10 g of each PPJ sample were dissolved in 100 mL of deionized water, let to stir at room temperature for 30 min, centrifuged and filtered as previously described.

#### 2.3.1. Total Polyphenols and Flavonoids

Total polyphenol content was determined using the Folin–Ciocalteau method as reported by Vazquez-Roncero et al. [15], with some modifications. An amount of the PPJ filtered sample (250 µL) was mixed with 1.25 mL of Folin–Ciocalteau reagent (FC) and allowed to react for 3 min, and then 2.5 mL of 20% sodium carbonate (Na_2_CO_3_) was added. The mixture was brought to a final volume of 25 mL and let to react in the dark for 1 h. After the incubation period, the absorbance was spectrophotometrically measured at 725 nm, using Perkin Elmer lambda 25 UV-Vis spectrometer, and the results were expressed on PPJ fresh weight (FW) as mg/100 g of gallic acid equivalents (mg GAE/100 g of FW).

The total flavonoid content was evaluated by using the aluminum chloride colorimetric method reported by Lin and Tang [16], with slight modifications. Ten grams of each PPJ sample were dissolved in 100 mL of deionized water, let to stir at room temperature for 30 min, centrifuged and filtered as previously described. An amount of 0.5 mL of this solution was then mixed with 1.5 mL of 95% alcohol, 0.1 mL of 10% aluminum chloride hexahydrate (AlCl_3_·6H_2_O), 0.1 mL of 1 M potassium acetate (CH_3_COOK) and 2.8 mL of deionized water. Blank was prepared by replacing the PPJ sample with deionized water. Each reaction mixture was incubated at room temperature for 40 min, and the absorbance was spectrophotometrically measured at 415 nm against the blank. Quercetin 3-β-d-glucoside was used as a standard to create a seven-point standard curve (0–50 mg/L) and results were expressed as quercetin equivalents (mg QE/100 g of FW). All the analyses were carried out in triplicate.

#### 2.3.2. Total Betalains

To evaluate betacyanin and betaxanthin content, 10 g of each PPJ sample were dissolved in 100 mL of deionized water, let to stir at room temperature for 30 min, centrifuged and filtered as previously described. The pigment amount extracted was spectrophotometrically analyzed [17,18] at 536 nm and 481 nm, for betacyanin and betaxanthin, respectively, using the molecular weight (Mw) and molar extinction coefficient (ε) in water of betanin (Mw = 550 g/mol; ε = 60,000 L/mol) and indicaxanthin (Mw = 308 g/mol; ε = 48,000 L/mol). The results were expressed as mg of betacyanin and betaxanthin on 100 g of sample (mg/100 g of FW). All the analyses were carried out in triplicate.

#### 2.3.3. DPPH Assay

PPJ filtered samples were analyzed for their antioxidant activity by using the DPPH radical scavenging activity method reported by Brand-Williams et al. [19], with slight modifications. The assay was conducted by reacting 3 mL of methanol DPPH (2,2-Diphenyl-1-picrylhydrazyl) solution 100 µM with 50 µL of the PPJ samples, homogenized and incubated in the dark for one hour at 25 °C. Control sample (blank) was prepared by replacing the same amount of juice with methanol. All the analyses were carried out in triplicate. At the end of the reaction period the absorbance of each sample was read at 515 nm using Perkin Elmer lambda 25 Uv-Vis spectrometer and the radical scavenging activity (RSA) was calculated according to the following Equation (1):

RSA (%) = [(Absorbance blank − Absorbance sample)/Absorbance blank] × 100(1)

### 2.4. Color Analysis

Color differences among PPJ samples were analyzed by using a portable colorimeter Konica Minolta CM-2500d (Bremen, Germany) equipped with a Light Protection Tube with plate 40 mm (CR-A33b) and a sample cell made of optical glass (Glass Cell CR-A504), using as illuminant D65. Each CIE L*a*b* color parameters defined in term of Lightness (L*), redness (a*) and yellowness (b*) space values and the psychometric correlates of chroma (C) and hue angle was expressed as the mean of 6 random reading ± standard deviation. Psychometric correlates of chroma (C) and hue angle (h) were calculated as previously reported [20].

The hue angle was expressed as Δh variation between “Agostani” and “Bastardoni” PPJ samples of the same cultivar. Moreover, among each cultivar the color differences between “Agostani” and “Bastardoni” PPJs was expressed as ΔE, which was calculated using the following Equation (2):

(2) $$\Delta E=\sqrt{\left[{\left(L_{x}-L_{0}\right)}^{2}+{\left(a_{x}-a_{0}\right)}^{2}+{\left(b_{x}-b_{0}\right)}^{2}\right]}$$

where subscript “x” indicates the color of “Agostani” and ‘0′ indicates the color of “Bastardoni”.

### 2.5. In Vitro Evaluation of Prickly Pear Juice Antimicrobial Activity

The in vitro antibacterial activity of each PPJ sample was evaluated by using the agar well diffusion test. Bacterial cultures, stored at 4 °C on Petri dishes containing Nutrient Agar (NA, CM0003, Oxoid, Basingstoke, UK), of *Escherichia coli*, *Salmonella enterica*, *Pseudomonas fluorescens* (Gram negative) and *Listeria innocua*, *Listeria gray*, *Staphylococcus aureus*, *Bacillus subtilis* and *Bacillus cereus* (Gram positive) strains, belonging to the Di3A (Dipartimento di Agricoltura, Alimentazione e Ambiente, University of Catania, Catania, Italy) collection, were grown in Nutrient Broth (NB, CM0001, Oxoid, Basingstoke, UK) at 37 °C (*E. coli*, *L. innocua*, *St. aureus* and *S. enterica*) or 30 °C (*B. subtilis*, *B. cereus* and *P. fluorescens*) for 24 h.

After the incubation period, each strain was individually inoculated into 20 mL of melted NA (cooled at 45 °C) to obtain a final concentration of 10^6^ cells/mL, rapidly vortexed and poured into sterile Petri plates. Once solidification, wells were made on agar plates by using a sterile cork borer (5 mm diameter) and filled with each PPJ sample, previously centrifuged at 10,000× *g* at 4 °C for 10 min in a centrifuge ALC 4239R and filtered with a 0.20 μm pore size membrane filter (Millipore^®^, Burlington, MA, USA). The plates were then incubated at the above mentioned temperatures for 48 h. In the control plates, the wells were filled only with sterile distilled water (SDW).

The inhibitory effect of the PPJ samples against target strains was assessed measuring the size (cm) of the inhibition zone (no bacterial growth) around the well. Each test was performed in triplicate.

### 2.6. Statistical Analysis

Statistical analyses were performed using the statistical package software Minitab™ version 16.0 (Minitab, LLC; State College, PA, USA). All data from experiments were expressed as mean values ± standard deviation. Data of different assays were analyzed independently and subjected to one-way analysis of variance (ANOVA). Fisher’s test was used to compare the significance of differences among means (*p* < 0.05).

## 3. Results and Discussion

### 3.1. Evaluation of Physical and Chemical Properties

Table 1 summarizes the physical and chemical characteristics of PPJs, obtained from “Agostani” and “Bastardoni” fruit of the three differently colored cultivars.

“Agostani” and “Bastardoni” exhibited an average pH of 6.37 ± 0.11, with the lowest value detected for “Agostani” fruits of the yellow cultivar Sulfarina that reached the pH value of 6.18 ± 0.05.

The total acidity was comprised between 0.019% and 0.026%; the significantly (*p* < 0.05) lowest content was detected only in the white cultivar of “Agostani” fruits (Table 1).

PPJ samples had an average soluble solid content of 12.88 ± 0.61 °Brix and, within the two harvest times (“Agostani” and “Bastardoni”), the yellow cultivar Sulfarina exhibited the significantly (*p* < 0.05) lowest value of 11.75 ± 0.25.

Reducing sugar of PPJ samples were in the range 11.05 ± 1.69–12.79 ± 2.28, with no statistically significant difference among samples.

Our findings were comparable to those reported by other researchers on differently colored prickly pear cultivars [21] and lower than those reported for Moroccan and Spanish prickly pears [22,23,24].

Protein (%) content of PPJs ranged from 1.22 to 1.74 g/100 g and it was significantly (*p* < 0.05) higher in “Bastardoni” fruits in comparison to “Agostani”. Such results were higher than those reported by Gurrieri et al., Dehbi et al. and El-Gharras et al. [21,22,23] and were comparable to those found by Díaz Medina et al., Sáenz and Sepúlveda and Silos-Espino et al. [24,25,26]. These differences may be attributed both to the pedoclimatic conditions at which prickly pears were grown and to the different variety.

### 3.2. Bioactive Compounds

**Figure 1:**
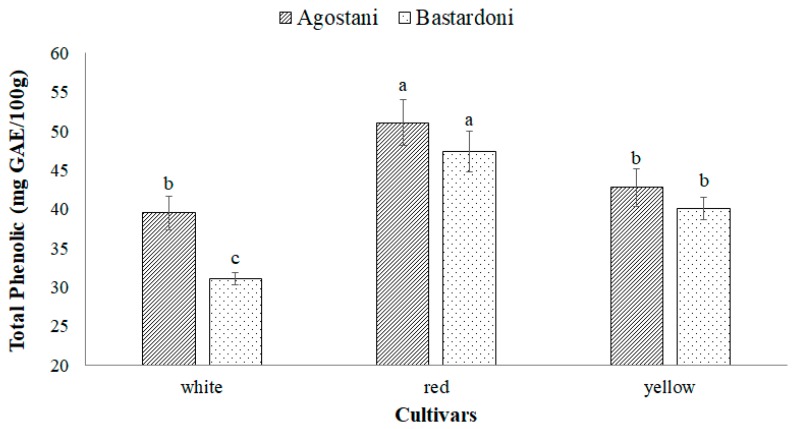
Total polyphenol content of prickly pear juices (PPJs), obtained from “Agostani” and “Bastardoni” fruit of the three differently colored cultivars (white: Muscaredda; red: Sanguigna; yellow: Sulfarina). Columns marked by different letters are significantly different according to Fisher’s least significant difference test (*p* < 0.05). Vertical bars indicate the standard deviation of the mean.

The total polyphenol content, displayed in Figure 1, evidenced as both “Agostani” and “Bastardoni” of the red cultivar Sanguigna registered the significantly (*p* < 0.05) highest values of 51.1 ± 2.9 and 47.4 ± 2.6 mg GAE/100 g, respectively, followed by the yellow cultivar Sulfarina (42.8 ± 2.4 and 40.1 ± 1.4 mg GAE/100 g, respectively for “Agostani” and “Bastardoni”). Although “Agostani” fruits registered a slightly higher value in comparison to “Bastardoni”, these differences were not significant (*p* > 0.05) in red and yellow cultivars.

“Agostani” fruits of the white cultivar Muscaredda showed a value not significantly (*p* > 0.05) different in comparison to the “Agostani” and “Bastardoni” yellow cultivar (39.5 ± 2.2 mg GAE/100 g), while “Bastardoni” of the white cultivar registered the significantly (*p* < 0.05) lowest value of 31.0 ± 0.82 mg GAE/100 g.

Overall, in our samples the content of polyphenols ranged from 31.0 ± 0.82 to 51.1 ± 2.9 mg GAE/100 g. These values indicated a good amount of phenols, considering that previous studies reported a content of phenolic compounds, in *Opuntia* spp. juice, ranging from a minimum of about 2.2 mg GAE/100 g to a maximum of about 66 mg GAE/100 g [22,24,27,28]. Moreover, the results obtained are partially in accordance with Cova et al. [7], who reported as all cultivars of “Agostani” had the highest polyphenol content in comparison to “Bastardoni”, although all samples registered averagely higher values in comparison to our samples. In contrast, they also reported as total polyphenols content of “Bastardoni” had no significant (*p* > 0.05) differences among the three colored cultivars.

**Figure 2:**
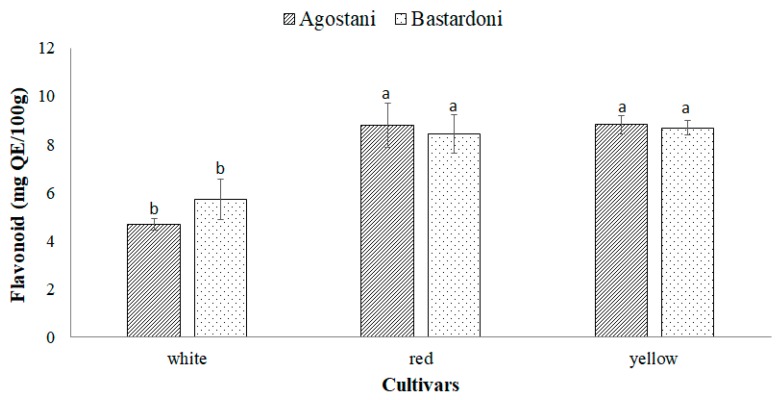
Total flavonoid content of prickly pear juices (PPJs), obtained from “Agostani” and “Bastardoni” fruit of the three differently colored cultivars (white: Muscaredda; red: Sanguigna; yellow: Sulfarina). Columns marked by different letters are significantly different according to Fisher’s least significant difference test (*p* < 0.05). Vertical bars indicate the standard deviation of the mean.

Regarding the total flavonoid content, displayed in Figure 2, both “Agostani” and “Bastardoni” PPJs of the red and yellow cultivars evidenced the average value of 8.7 ± 0.17 mg QE/100 g. Although the content of flavonoids was slightly higher in “Agostani” samples than in “Bastardoni” ones (Figure 2) the differences were not significant (*p* > 0.05). In comparison to red and yellow cultivars, both “Agostani” and “Bastardoni” of the white cultivar showed the significantly (*p* < 0.05) lowest flavonoid content of 4.7 ± 0.3 and 5.7 ± 0.8 mg QE/100 g, respectively (Figure 2).

**Figure 3:**
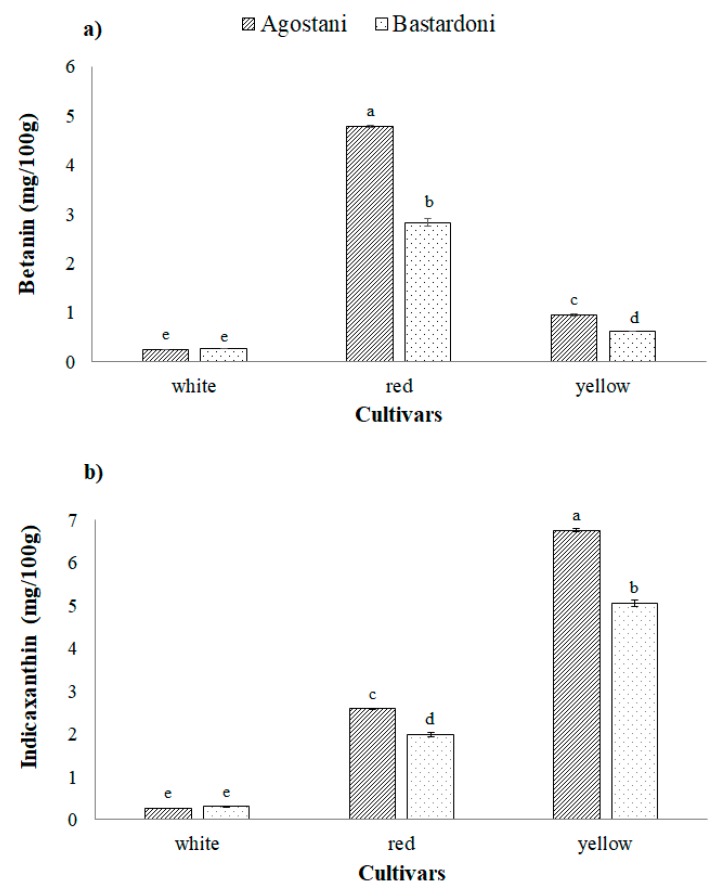
Total betanin (**a**) and indicaxanthin (**b**) content of prickly pear juices (PPJs), obtained from “Agostani” and “Bastardoni” fruit of the three differently colored cultivars (white: Muscaredda; red: Sanguigna; yellow: Sulfarina). Columns marked by different letters are significantly different according to the Fisher’s least significant difference test (*p* < 0.05). Vertical bars indicate the standard deviation of the mean.

Figure 3a,b reports the total content of betanins and indicaxanthin evaluated on white, red and yellow cultivars of “Agostani” and “Bastardoni” crop productions.

Betanins (Figure 3a) were mostly represented in the red cultivars; in fact, the significantly (*p* < 0.05) highest value was detected in red “Agostani” fruits (4.8 ± 0.01 mg/100 g), followed by red “Bastardoni” (2.8 ± 0.01 mg/100 g). Furthermore, “Agostani” fruits of the yellow cultivar possessed a significantly higher (1.0 ± 0.02 mg/100 g) amount of betanin, if compared to the yellow cultivar of “Bastardoni” crop production (0.6 ± 0.00 mg/100 g). White cultivar registered the lowest values (0.2 ± 0.01 and 0.3 ± 0.01 mg/100 g for “Agostani” and “Bastardoni”, respectively) with no significant differences (*p* > 0.05) between the two crop productions.

The content of indicaxanthin was evidently high in the yellow cultivar and, within it, “Agostani” fruits showed the significantly highest value (6.8 ± 0.04 mg/100 g), followed by “Bastardoni” ones (5.1 ± 0.08 mg/100 g; Figure 3b). Moreover, also within the red cultivar, “Agostani” fruits exhibited a significantly (*p* < 0.05) higher value of indicaxanthin (2.6 ± 0.02 mg/100 g), if compared with “Bastardoni” (2.0 ± 0.06 mg/100 g). No significant differences were detected between the two crop productions of the white cultivar, which showed the lowest values (both of 0.3 ± 0.01 mg/100 g).

Betanin and indicaxanthin contents determined on PPJ of Sicilian fruits was similar to those reported both for Italian and Mexican samples [28,29] and generally lower than those studied by Stintzing et al. in California (USA) [27].

**Figure 4:**
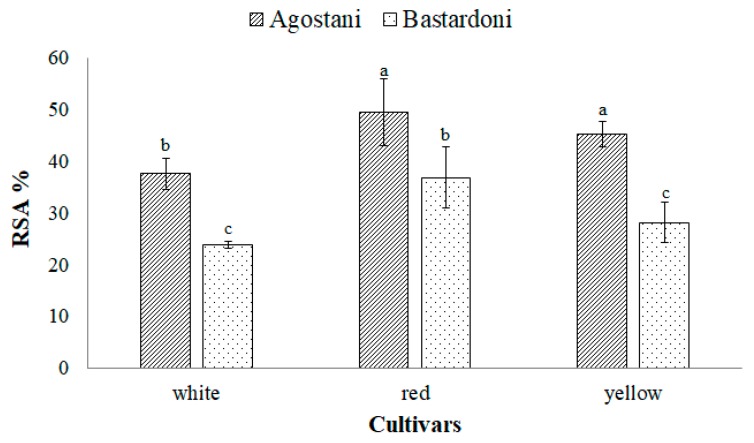
DPPH inhibition (%) of prickly pear juices (PPJs), obtained from “Agostani” and “Bastardoni” fruit of the three differently colored cultivars (white: Muscaredda; red: Sanguigna; yellow: Sulfarina). Columns marked by different letters are significantly different according to Fisher’s least significant difference test (*p* < 0.05). Vertical bars indicate the standard deviation of the mean.

The determination of antioxidant activity on PPJ samples, displayed in Figure 4, evidenced as “Agostani” crop production of the red and yellow cultivars showed the significantly (*p* < 0.05) highest values in free-radical scavenging activity, 49.4 ± 2.46 and 45.2 ± 6.45 RSA%, respectively. Within “Agostani” crop production, the white cultivar registered the lowest value of 37.6 ± 2.98, which was not significantly different (*p* > 0.05) from that recorded in “Bastardoni” of the red cultivar (36.8 ± 5.85), which instead represented the highest value.

The significantly (*p* < 0.05) lowest values of RSA % were recorded in white and yellow cultivars of “Bastardoni” crop production (23.8 ± 0.6 and 28.1 ± 3.9, respectively).

The antioxidant proprieties of betalain have been demonstrated to be three fold stronger than that of ascorbic acid [30] and in many studies its high content and the high content of polyphenols was correlated with a good antioxidant activity [31,32,33]. The association between the content of these compounds and the antioxidant activity has also been highlighted by Yahia and Mondragon-Jacob [34], who evidenced as in a yellow variety of *O. albicarpa*, which did not contain betalain and with a low content of phenolic compounds, the antioxidant activity was really low.

In this study, the red and yellow cultivars with great content in betalain showed the highest value of RSA%. Moreover, “Agostani” cultivars evidenced the highest antioxidant activity in comparison to “Bastardoni”, probably due to the highest amount of polyphenols and flavonoids, although the differences were not always significant.

### 3.3. Color Evaluation

Color values of PPJ samples of white, red and yellow cultivars belonging to “Agostani” and “Bastardoni” crop productions were compared and reported in Table 2.

Both white cultivar of “Agostani” and “Bastardoni” registered the significantly (*p* > 0.05) highest L* values followed by the yellow and red cultivar. Within each cultivar (white, red and yellow) the “Agostani” fruit always displayed a significantly (*p* > 0.05) higher value of L* in comparison to “Bastardoni”.

Red and yellow cultivars presented higher values of a* than white cultivar, while b* values were the highest in white cultivar and lowest in the red one. This trend is due to the fact that the color of prickly pear fruit is strongly influenced by the presences of indicaxanthin and betacyanin content, each of which led to a variation from yellow to red, depending on the amount in the matrix, and therefore determined the overall fruit color [27].

The C value depended on the relative ratios of betaxanthins and betacyanins and measuring color saturation or intensity showed higher values in white cultivar, followed by yellow cultivar and among them the significant (*p* < 0.05) highest values were always recorded for “Bastardoni.”

The recorded h value, representing the hue angle and describing the relative amounts of redness and yellowness, was significantly (*p* < 0.05) higher in white and yellow cultivar, followed by red cultivar; among cultivars, “Bastardoni” evidenced the highest h value.

To better evaluate the differences between each cultivar (white, red and yellow) of “Agostani” and “Bastardoni”, the ∆E value was calculated, and differences were considered “very distinct” if ∆E > 3, “distinct” if 1.5 < ∆E < 3 and “without perceptible differences” if ∆E < 1.5, as reported by Francis and Clydesdale [35].

In our study, very distinct differences were observed among “Agostani” and “Bastardoni” samples of white and yellow cultivars, both of which registered ∆E > 3. Red cultivar evidenced less pronounced differences but always discernible to the naked eye, recording a ∆E of 2.21 ± 1.06.

### 3.4. Antimicrobial Activity of Ppjs Against Target Bacteria

“Agostani” and “Bastardoni” of the three cultivars (white, red and yellow) were tested for their potential antimicrobial activity, through the agar well diffusion method on NA, against the nine pathogens mentioned above.

As reported in Table 3, all samples revealed similar and remarkable antibacterial activity against *S. enterica*, and a modest activity towards *Ps. fluorescens*. In particular, this latest was more inhibited by both white and red cultivars of “Agostani” and “Bastardoni” and by the yellow cultivar of “Bastardoni”, which produced the significantly (*p* < 0.05) highest inhibition halo. Cell growth of *E. coli* was also inhibited by PPJs tested, except for those obtained by the white cultivar that evidenced a lack of activity, regardless harvest time.

Among Gram-positive species, only *B. subtilis* was effectively inhibited by the PPJ samples tested, regardless cultivar and harvest time (Table 3).

To the best of our knowledge, no researches investigated the potential antimicrobial activity of prickly pear juice or semi-processed juice, while many authors reported antimicrobial effects obtained by the extracts from various parts of the plant [36,37,38].

The antimicrobial activity of *Opuntia* fruit extract was correlated with the antioxidant activity and the betalain pigment content [39]. Many authors reported that the fruit extract obtained by using different technique and different solvents showed an antimicrobial activity against a broad spectrum of bacteria [40,41,42,43] and fungi [41].

In our study, antimicrobial activity against *S. enterica* and *Ps. fluorescens* has been exerted by almost all the PPJ samples, including those deriving from the white cultivar, suggesting that the antibacterial activity against these species is probably mainly due to the high presence of polyphenols. Such efficacy against Gram-negative bacterial species is of particular interest since most of the research studies have shown that Gram-positive bacteria are more susceptible towards plant extracts as compared to Gram negative ones [44,45,46,47]. The sensitivity to PPJ samples of targeted bacteria evinced in this study broadened the range of natural compounds able to act as food preservatives in replacement of the chemical ones.

## 4. Conclusions

The PPJs both obtained from “Agostani” and “Bastardoni” fruit can be considered as natural sources of bioactive compounds with marked antioxidant activity. The juices from “Agostani” fruits had a higher content of polyphenols and betalains and antioxidant activity than the juice made from “Bastardoni” fruits. Therefore, “Agostani” fruits, considered as an industrial by-product, could be processed as a promising ingredient for the enrichment of nutritional properties and antioxidant capacity of finished food products. Moreover, a good antimicrobial activity against target bacteria was exerted by all analyzed juice samples, including the one obtained by the white cultivar *Muscaredda*, which contains the lowest levels of total phenolics and betalain pigments, can be considered a suitable ingredient in a wider range of food application.

## Figures and Tables

**Table 1:** Physical and chemical characteristics of prickly pear juices (PPJs), obtained from “Agostani” and “Bastardoni” fruit of the three differently colored cultivars (white: Muscaredda; red: Sanguigna; yellow: Sulfarina).

		pH	Citric Acid (%)	°Brix	Reducing Sugar (%)	Dry Matter (%)	Protein (%)
**Agostani**	white	6.30 ± 0.03 d	0.019 ± 0.000 b	13.00 ± 0.00 a	12.73 ± 1.05 a	13.99 ± 0.23 ab	1.24 ± 0.01 b
	red	6.43 ± 0.03 b	0.026 ± 0.000 a	13.25 ± 0.75 a	12.79 ± 2.28 a	12.85 ± 0.12 ab	1.22 ± 0.05 b
	yellow	6.18 ± 0.05 e	0.026 ± 0.000 a	11.75 ± 0.25 b	11.05 ± 1.69 a	12.48 ± 1.36 b	1.23 ± 0.24 b
**Bastardoni**	white	6.50 ± 0.03 a	0.026 ± 0.000 a	12.70 ± 0.75 a	11.80 ± 1.21 a	14.10 ± 1.97 a	1.65 ± 0.40 a
	red	6.40 ± 0.03 c	0.022 ± 0.003 ab	13.50 ± 0.50 a	12.60 ± 1.11 a	13.36 ± 0.53 ab	1.74 ± 0.14 a
	yellow	6.40 ± 0.02 b	0.0026 ± 0.006a	13.00 ± 0.00 a	11.50 ± 1.12 a	14.05 ± 0.93 a	1.65 ± 0.18 a

Data presented as mean ± standard error of the mean. In each column, values followed by different letter within the same parameter are significantly different according to the Fisher’s least significant difference test (*p* ≤ 0.05).

**Table 2:** Color parameters of prickly pear juices (PPJs), obtained from “Agostani” and “Bastardoni” fruit of the three differently colored cultivars (white: Muscaredda; red: Sanguigna; yellow: Sulfarina).

Samples		Color Parameters						
		L*	a*	b*	C	h	ΔE	Δh
White	Agostani	58.52 ± 0.53 a	−8.43 ± 0.26 e	55.95 ± 0.75 a	56.58 ± 0.76 a	98.57 ± 0.22 b	11.13 ± 0.98 a	−2.42 ± 0.54 b
	Bastardoni	48.60 ± 0.69 b	−9.94 ± 0.42 f	51.19 ± 0.81 b	52.15 ± 0.81 c	100.99 ± 0.48 a		
Red	Agostani	10.53 ± 0.42 e	25.72 ± 0.56 c	10.36 ± 0.25 d	27.73 ± 0.58 e	21.94 ± 0.44 e	2.21 ± 1.06 c	−0.02 ± 0.04 a
	Bastardoni	9.53 ± 0.68 f	24.16 ± 0.64 d	10.22 ± 0.95 d	26.25 ± 0.40 f	22.94 ± 2.35 e		
Yellow	Agostani	25.74 ± 0.37 c	39.87 ± 0.61 a	34.97 ± 0.34 c	53.04 ± 0.53 b	41.25 ± 0.49 d	7.53 ± 0.78 b	−0.10 ± 0.01 a
	Bastardoni	24.59 ± 0.97 d	32.57 ± 0.67 b	35.04 ± 0.86 c	47.84 ± 0.74 d	47.09 ± 0.97 c		

Data presented as mean ± standard error of the mean. In each column, values followed by different letter within the same parameter are significantly different according to the Fisher’s least significant difference test (*p* ≤ 0.05).

**Table 3:** Antimicrobial activity of prickly pear juices (PPJ), obtained from “Agostani” and “Bastardoni” fruit of the three differently colored cultivars (white: *Muscaredda*; red: *Sanguigna;* yellow: *Sulfarina*). Data are reported as the width of the inhibition halo (cm) produced by the PPJ sample against target bacterial species.

	Agostani			Bastardoni		
	White	Red	Yellow	White	Red	Yellow
*E. coli*	-	0.60 ± 0.00 a	0.60 ± 0.00 a	-	0.50 ± 0.00 b	0.60 ± 0.00 a
*S. enterica*	1.00 ± 0.00 a	0.97 ± 0.06 a	0.97 ± 0.06 a	1.00 ± 0.00 a	1.00 ± 0.00 a	0.97 ± 0.06 a
*Ps. fluorescens*	0.23 ± 0.03 a	0.10 ± 0.00 c	-	0.27 ± 0.03 a	0.17 ± 0.03 b	0.27 ± 0.03 a
*L. innocua*	-	-	-	-	-	-
*L. gray*	-	-	-	-	-	-
*St. aureus*	-	-	-	-	-	-
*B. subtilis*	0.77 ± 0.06 a	0.27 ± 0.06 c	0.20 ± 0.00 c	0.27 ± 0.06 c	0.47 ± 0.06 b	0.50 ± 0.00 b
*B. cereus*	-	-	-	-	-	-

Data presented as mean ± standard error of the mean. In each row, values followed by different letter are significantly different according to Fisher’s least significant difference test (*p* ≤ 0.05).
